# Visual and Non-Visual Navigation in Blind Patients with a Retinal Prosthesis

**DOI:** 10.1371/journal.pone.0134369

**Published:** 2015-07-30

**Authors:** Sara Garcia, Karin Petrini, Gary S. Rubin, Lyndon Da Cruz, Marko Nardini

**Affiliations:** 1 Institute of Ophthalmology, University College London (UCL), London, United Kingdom; 2 Department of Psychology, University of Bath, Bath, United Kingdom; 3 (NIHR) Biomedical Research Centre at Moorfields Eye Hospital and UCL Institute of Ophthalmology, London, United Kingdom; 4 Vitreoretinal Service, Moorfields Eye Hospital NHS Foundation Trust, London, United Kingdom; 5 Department of Psychology, Durham University, Durham, United Kingdom; University of Montreal, CANADA

## Abstract

Human adults with normal vision can combine visual landmark and non-visual self-motion cues to improve their navigational precision. Here we asked whether blind individuals treated with a retinal prosthesis could also benefit from using the resultant new visual signal together with non-visual information when navigating. Four patients (blind for 15-52 years) implanted with the Argus II retinal prosthesis (Second Sight Medical Products Inc. Sylmar, CA), and five age-matched and six younger controls, participated. Participants completed a path reproduction and a triangle completion navigation task, using either an indirect visual landmark and non-visual self-motion cues or non-visual self-motion cues only. Control participants wore goggles that approximated the field of view and the resolution of the Argus II prosthesis. In both tasks, control participants showed better precision when navigating with reduced vision, compared to without vision. Patients, however, did not show similar improvements when navigating with the prosthesis in the path reproduction task, but two patients did show improvements in the triangle completion task. Additionally, all patients showed greater precision than controls in both tasks when navigating without vision. These results indicate that the Argus II retinal prosthesis may not provide sufficiently reliable visual information to improve the precision of patients on tasks, for which they have learnt to rely on non-visual senses.

## Introduction

During navigation, humans with healthy vision rely on both visual and non-visual sensory information to update their position and orientation within their environment. Like other mammals, humans can use both visual landmarks and idiothetic self-motion cues (including those from vestibular and proprioceptive sensory systems) to track their own movements over time [1–4]. Research has also shown that adults with healthy vision can improve their navigational performance by combining visual and non-visual cues [5–8]. In many of these studies, improvements in navigation precision were well predicted by an ideal observer model that averages visual and non-visual sensory estimates, weighted by their reliability.

Following visual loss, individuals must rely solely on non-visual sensory information to make perceptual judgments, and consequently this may impact their performance on many everyday multisensory tasks like navigation. Blind individuals are no longer able to use visual information in combination with non-visual information, although they could still in principle combine information from different non-visual modalities to reduce their sensory estimate uncertainty [9]. They can also use sensory substitution devices to perceive visual information converted into different non-visual signals [10, 11]. Furthermore, research has documented enhanced perception by non-visual modalities for certain tasks following blindness, including tactile discrimination [12, 13], spatial sound localization [14, 15] and spatial route learning [16]. This improvement in the reliability of non-visual processing has been linked to cross-modal neural reorganisation, with studies showing non-visual recruitment of the occipital cortex in the blind (e.g. review by [17, 18]). Therefore, whilst the loss of vision may be expected to lead to increased uncertainty and impoverished performance in multisensory activities, this may be partly counterbalanced by a potential reorganisation of sensory processing. Such compensatory plasticity has implications for treatments that aim to restore vision by stimulating the deprived visual system directly, such as retinal prostheses.

**Fig 1:**
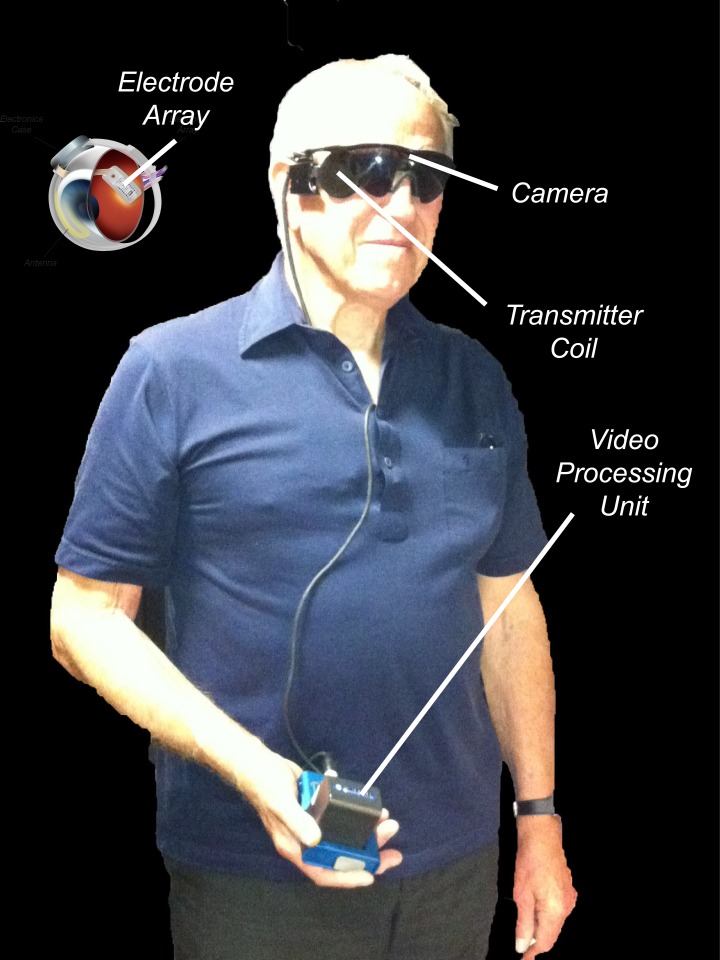
The Argus II retinal prosthesis system (Second Sight Medical Products, Inc., Sylmar, CA). Used with permission from Second Sight Medical Products. The system consists of: (i) a pair of glasses fitted with a miniature video camera (510 x 492 resolution) for external image capture, (ii) an external Video Processing Unit (VPU) that processes the video signal into a 10 x 6 pixel image, matching the array’s field of view, and (iii) a 6 x 10 electrode epiretinal array (18 x 11 degrees) secured over the fovea.

The Argus II retinal prosthesis system (Second Sight Medical Products, Inc., Sylmar, CA) is a new treatment that aims to restore vision to patients blinded by outer retinal degenerative diseases, such as retinitis pigmentosa [19]. The device consists of a glasses-mounted miniature camera that sends live video data to an externally-worn processing unit that transforms it into electrical stimulation patterns. These patterns are sent wirelessly to an implant on the retina, directly stimulating preserved retinal cells (see Fig 1). Using the Argus II system, patients who have been visually deprived for a number of years are once again able to receive visual input. However, prosthetic vision is different to native vision and patients have to learn to interpret the pixelated phosphenes elicited by the implant. Furthermore, the direction of ‘gaze’ is defined by head position and not by eye position, and due to the small field the environment is explored by head scanning.

In this study we examine whether blind patients, implanted with the Argus II retinal prosthesis, are able to use this new visual signal together with non-visual information, to improve their performance on two well-known navigation tasks: a path reproduction and a triangle completion task. Although previous research has shown that normally sighted adults are able to combine vision with other sensory information to improve their precision [5–8], it is unknown whether patients, who have been visually-deprived for a number of years, will similarly benefit from using the new visual information afforded by the prosthesis with other sensory information. To this end, we used two tasks for which both visual information, about an indirect landmark, and non-visual (i.e. vestibular and proprioceptive) self-motion information were potentially useful for improving performance. We assessed whether participants improved their navigational precision or accuracy when given visual and non-visual self-motion cues together, compared to when using non-visual information alone.

## Methods

### Ethics Statement

Patients were recruited from the Moorfields Eye Hospital NHS Foundation Trust, London, UK, and the study had received ethical approval from the East Central London committee. Ethical approval for conducting the study with control adults was received from the research ethics board of University College London. Informed written consent, according to the Tenets of the Declaration of Helsinki, was obtained from all participants prior to participation.

### Participants

Four patients (3 male; aged 49–77 years; mean age 66.0 years) implanted with the Argus II prosthesis in their right eye, at Moorfields Eye Hospital, 4–7 years prior to testing, participated. All had been diagnosed with a retinal degenerative disease (3 retinitis pigmentosa, 1 choroideremia) prior to implant, and reported having been blind for 15–52 years. All patients had some level of bare light perception, but no measurable visual acuity (logMAR 2.9, bare light perception or worse in both eyes). Patients had received visual rehabilitation training provided by Second Sight Inc. prior to this study, that covered basic skills like head scanning. Six young adults (aged 23–29 years; mean age 25.7 years) and 5 age-matched adults (aged 54–74 years; mean age 63.0 years), all with normal or corrected vision, also participated in this study.

### Procedure

Participants were asked to complete two tasks: path reproduction and triangle completion. Both tasks were conducted in a darkened room (6.5m x 7.75m), with black walls and black carpet, and involved using a single landmark: an illuminated white square paper shade floor lamp (0.23m x 0.23m x 1.52m, 200cd/m^2^ (lamp) against 0.04cd/m^2^ (walls & carpet)).

#### Path reproduction task

**Fig 2:**
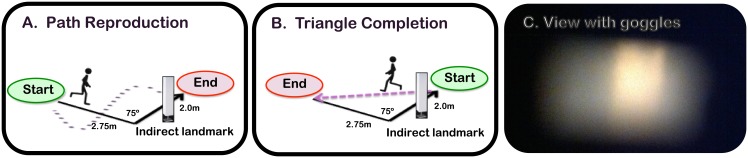
A & B: Schematic of path reproduction and triangle completion tasks. C: View of the landmark through the goggles worn by normally sighted participants. Participants were guided along the black path by the experimenter, and then: (i) For path reproduction, guided back to the start position and asked to reproduce the path as accurately as possible. (ii) For triangle completion, asked to return to the start position as accurately as possible.

Participants were led to a start position and advised that the experimenter would guide them along a path which they would then be asked to reproduce as accurately as possible. The path comprised of an initial 2.5m leg, a 75^°^ rotation, and a final 2m leg. The landmark was positioned midway along the second leg. This meant that it could potentially provide information about the correct initial heading, the distance after which to turn, and the correct turning angle, (see Fig 2A).

#### Triangle completion task

As for the path reproduction task, participants were led to a start position and guided by the experimenter along an outbound path, comprising of an initial 2.5m leg, a 75^°^ rotation, and a final 2m leg. However, in this task, participants were asked to return directly to the start position as accurately as possible on reaching the end of the outbound path, thereby completing a walked triangle (see Fig 2B). The landmark position was the same as for the path reproduction task. It could potentially provide information about the correct return turning angle.

Patients were asked to complete both tasks using (i) the retinal prosthesis (i.e. system on) and (ii) no vision (i.e. blindfolded and landmark light off). Three patients able to locate the landmark using their residual vision also completed the task with the system off (see S1 Table). Control (normally sighted) participants were asked to wear goggles that restricted their field of view (18 x 11 degrees) and visual resolution (1.6 logMAR), and similarly completed both tasks using (i) restricted vision and (ii) no vision (i.e. blindfolded and landmark light off).

Control participants were used to establish whether similarly restricted vision (in terms of field of view and resolution) could provide useful information about the visual landmark to individuals who would usually rely on vision for navigation. This would allow us to exclude the possibility that any failure of patients to use the landmark simply reflects its limited field of view and spatial resolution for these tasks. Additionally, we were able to compare control participant and patient performance when navigating without vision, so as to assess whether patients’ long-term visual deprivation may have led them to develop improved non-visual navigation skills.

To ensure patients were able to localise the visual landmark, all were initially asked to walk directly toward the landmark from ten different locations within the room (differing in distance and angle from the landmark) with the system on. All four patients were able to complete this task from all ten locations. They then completed the path reproduction task first, followed by the triangle completion task. Condition order within tasks was random, and each participant completed two blocks of five trials per condition.

### Data Acquisition & Analysis

Participants’ positions were tracked using an optical tracking system (8 Vicon MX13 cameras) through the monitoring of five helmet-mounted reflective markers. Position coordinates were saved using Vizard (Version 4.0; Santa Barbara, CA: WorldViz LLC.) and analysed using Matlab (Version R2014a, The MathWorks, Inc., Natick, Massachusetts, United States) and Psychtoolbox command library [20, 21]. A bivariate normal distribution was fitted to each participant’s end positions (i.e. where participants decided to stop), to estimate the x mean, y mean, x variance, y variance and x-y covariance for each condition. The FASTMCD algorithm [22], as implemented in the Libra toolbox for Matlab [23] was used to estimate these values robustly, with the assumption of 1% aberrant (outlier) values (i.e. a value of 0.99 for the alpha parameter). The sum of the variance in x and y directions was used to obtain a single measure of total variable error, reflecting the uncertainty (or imprecision) of spatial estimates. Variable error is expected to reduce when more precise information is available, or when information from multiple sources is averaged [24, 25]. Additionally, a measure of constant error was calculated as the distance between the correct end location and the participant’s end position. Constant error reflects a systematic bias (or inaccuracy) in spatial estimation, and is expected to reduce when a less biased information source is available.

Paired samples t-tests were run on control data to test for significant reductions in errors when using vision compared to when navigating without vision. Where vision was found to improve performance on navigational tasks, improvements were quantified as follows: The improvement in precision when using vision (restricted/prosthesis), was calculated as the difference in variable error when navigating with vision, compared to when navigating without vision. The improvement in accuracy when using vision (restricted/prosthesis) was calculated as the difference in constant error with vision, compared to when navigating without vision.

Given the small number of patients tested, we have elected not to report p values from inferential statistical tests. Instead, we compare each patient’s performance to the 95% confidence intervals calculated from participants with normal vision. Patient data falling outside the confidence limits indicates that the difference is unlikely to have resulted from measurement error alone.

## Results

### Differences in error between young and age-matched controls

A one-way ANOVA indicated no statistically significant differences in variable errors or constant errors between young and age-matched participants in the path reproduction or triangle completion tasks, (see Table 1). Consequently, control data was pooled together for further analysis.

**Table 1:** Results of a one-way ANOVA comparing variable and constant errors between young and age-matched participants.

	Variable Error		Constant Error	
	Path Reproduction	Triangle Completion	Path Reproduction	Triangle Completion
**Vision**	*F*[1,9] = 0.003, *p* = 0.958	*F*[1,9] = 1.070, *p* = 0.328	*F*[1,9] = 2.076, *p* = 0.183	*F*[1,9] = 0.020, *p* = 0.890
**No Vision**	*F*[1,9] = 2.199, *p* = 0.17	*F*[1,9] = 0.429, *p* = 0.529	*F*[1,9] = 0.548, *p* = 0.478	*F*[1,9] = 3.500, *p* = 0.094

### Variable Error

#### Path reproduction

**Fig 3:**
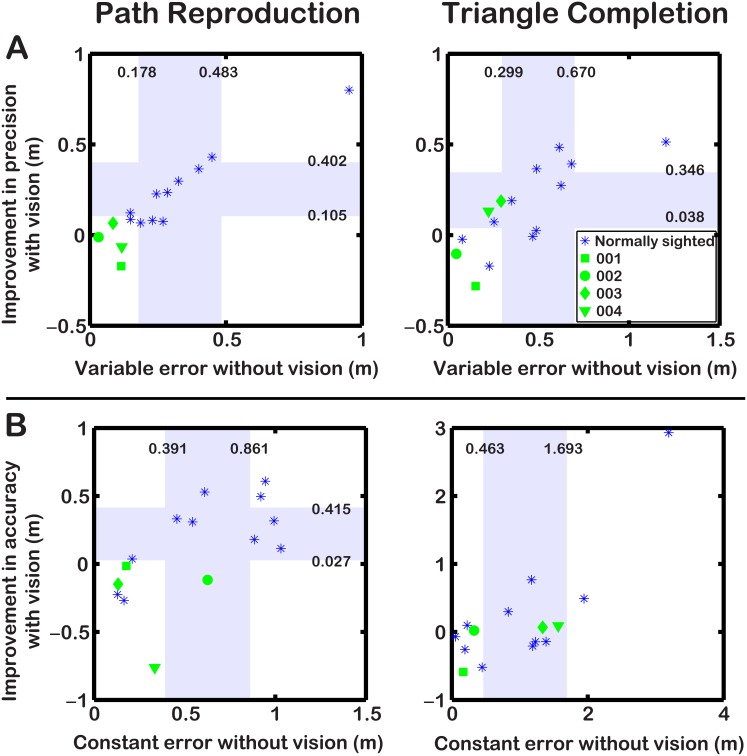
Graph showing improvement in variable error (A) or constant error (B) when using vision against errors when navigating without vision. Shading indicates the 95% confidence intervals computed from the control data. Path Reproduction: Patients did not show similar improvements in precision or accuracy when navigating with the prosthesis as controls. All had lower variable errors without vision, and three had lower constant errors without vision, compared to controls. Triangle Completion: Two of four patients showed similar improvements in precision when using vision as controls, and all patients showed lower variable errors without vision compared to controls. Two patients had lower constant errors without vision compared to controls.

A paired samples t-test indicated that control participants had significantly higher variable errors without vision than with vision (*t*[10] = 3.806, *p* = 0.003). Based on these control data, 95% of normally sighted participants would be expected to show reductions in error of 0.105m–0.402m. Patient data fell outside of these confidence intervals, and three of four showed better performance *without* vision. In addition, all four patients’ variable errors without vision were less than the lower limit of the 95% confidence intervals of normally sighted participants (ID 001–004: 0.115m, 0.031m, 0.084m, 0.117m compared to 95% CI: 0.178m–0.483m; see Fig 3A).

#### Triangle completion

A paired samples t-test indicated that control participants had significantly higher variable errors without vision than with vision (*t*[10] = 2.780, *p* = 0.020). Based on these control data, 95% of normally sighted participants would be expected to show reductions in error of 0.038m–0.346m. Two patients showed data that fell outside of these confidence intervals, showing better performance *without* vision. Again, all four patients’ variable errors without vision were less than the lower limit of the 95% confidence intervals of normally sighted participants (ID 001–004: 0.153m, 0.047m, 0.294m, 0.225m compared to 95% CI: 0.299m–0.670m; see Fig 3A).

### Constant Error

#### Path reproduction

A paired samples t-test indicated that control participants had significantly higher constant errors without vision than with vision (*t*[10] = 2.537, *p* = 0.030). Based on this control data, 95% of normally sighted participants would be expected to show reductions in error of 0.027m–0.415m. Patient data fell outside of these confidence intervals, and all patients showed better performance *without* vision. Three of four patients’ constant errors without vision were less than the lower limit of the 95% confidence intervals of normally sighted participants (ID 001, 002, 004: 0.178m, 0.133m, 0.335m compared to 95% CI: 0.391m–0.861m; see Fig 3B).

#### Triangle completion

A paired samples t-test indicated that control participants showed no significant differences in constant errors without vision than with vision (*t*[10] = 1.0316, *p* = 0.3266). Two of four patients’ constant errors without vision were less than the lower limit of the 95% confidence intervals of normally sighted participants (ID 003, 004: 0.169m, 0.327m compared to 95% CI: 0.463m–1.693m; see Fig 3B).

A Spearman’s correlation was used to test whether any inability to improve performance when using vision could be due to floor effects (i.e. participants having already very precise and accurate non-visual performance). There were significant positive associations between errors in the no vision condition and the percentage improvement in errors with vision, for both tasks, indicating that participants with greater errors in the no vision condition showed greater improvements with vision (Variable errors: path reproduction: *r*_*s*_[13] = 0.70, *p* = 0.005; triangle completion: *r*_*s*_[13] = 0.58, *p* = 0.027; Constant errors: path reproduction: *r*_*s*_[13] = 0.55, *p* = 0.038; triangle completion: *r*_*s*_[13] = 0.52, *p* = 0.049). This shows that more precise/accurate non-visual navigators had limited potential to improve with vision.

### Learning Effect

As the same path was repeated, to understand whether participants learnt over the course of the experiment, linear regression analyses were run to assess (i) the effect of trial number on constant error and (ii) the effect of block number on variable error. Blocks rather than trials were used for variable error because variance can only be calculated over a set of trials. Trial number did not statistically significantly predict constant error in either path reproduction or triangle completion tasks for control or patient participants (path reproduction: controls: *F*[1,28] = 0.140, *p* = 0.712; patients: *F*[1,28] = 3.258, *p* = 0.082; triangle completion: controls: *F*[1,28] = 0.194, *p* = 0.663; patients: *F*[1,28] = 0.594, *p* = 0.447). Similarly, block number did not statistically significantly predict variable error in either task for either group (path reproduction: controls: *F*[2,4] = 0.006, *p* = 0.941; patients: *F*[2,4] = 2.040, *p* = 0.227; triangle completion: controls: *F*[2,4] = 1.070, *p* = 0.360; patients: *F*[2,4] = 0.927, *p* = 0.390). No significant effect of trial number on constant error, or block number on variable error, within conditions was found (see S4 Table). These results indicate that participants did not learn over the course of this experiment. If participants had shown learning, it would have been necessary to consider the effect of task order and any differing learning rates between groups, when comparing their navigational errors.

## Discussion

This study assessed whether patients implanted with the Argus II retinal prosthesis would use this new visual signal to improve navigational precision, by using spatial information provided by an indirect visual landmark (an illuminated floor lamp) as well as non-visual self-motion cues. Low resolution, restricted field of view vision was sufficiently informative to lead to improvements in navigational precision in normally sighted participants, in both a path reproduction and triangle completion task. In a multisensory cue integration framework [24, 25], this shows that the visual cue was at least as useful (reliable), for normally sighted adults, as non-visual self-motion information. Two patients implanted with the Argus II prosthesis showed similar improvements in precision (to normally sighted adults) on a triangle completion task. In contrast, three patients on the path reproduction task and two patients on the triangle completion task showed reduced precision when navigating with the Argus II prosthesis. This result is consistent with patients either (i) using the (more variable) visual cue only (ii) switching between visual and non-visual cues or (iii) combining visual and non-visual information, but not weighting these according to their reliability.

Argus II patients’ inability to use the visual landmark to improve their navigational precision is partly due to dissimilarities between the new vision afforded by the prostheses and the native vision (even when restricted by goggles): Firstly, the field of view and resolution of the Argus II were more limited for some patients than that approximated by the control-worn goggles. Both field of view and resolution are dependent on the number of functioning electrodes in the implant, and only one patient had all electrodes working (this varied among the four patients, from 47–100%). Secondly, the vision afforded by the prostheses consists of pixelated phosphenes and thus is qualitatively different to the blurred vision experienced by control participants. Thirdly, because the phosphenes elicited by the device have variable persistence independent of the stimulus [26], using this visual information sometimes requires memory and/or continual head scanning to elicit further phosphenes. Despite differences between native and prosthetic vision, all four patients were able to perceive the landmark, as shown by their ability to walk directly toward it from various locations within the room. However, obtaining accurate estimates of angles and distances using prosthetic vision was challenging for them. Patients reported using their head scanning movements together with the percepts elicited to estimate angles and distances. For example, they computed the landmark’s distance by considering the amount they had to move their head to detect its edges (small head scanning movements being sufficient from far distances, larger head scanning movements needed if near to the landmark). These computations are effortful and subject to inaccuracies. Indeed, in the path reproduction task, controls showed significantly lower constant errors when navigating with vision, compared to without, but patients showed higher mean constant errors with the prosthesis instead, indicating that the prosthesis tended to bias their navigation estimates.

In addition, to limitations in the signal afforded by the prosthesis, differences between groups in non-visual processing may also partly account for the differential results between groups and across conditions. Specifically, patients showed lower variable errors when navigating without vision, compared to controls, in both path reproduction and triangle completion tasks. Results showed that the degree of improvement in error when navigating with vision was positively associated with the magnitude of error when navigating without vision. Thus, participants who were already very precise non-visual navigators were limited in their potential to improve their performance with vision. Path reproduction can be done by accurate encoding of distances and turns via self-motion [27], while in triangle completion participants must compute angles and distances so as to decide on a *new* (previously not walked) home-bound path [8]. Triangle completion can be done by self-motion alone [28], but visual landmarks can usefully reduce errors, and predominate over self-motion in healthy adults [29]. Correspondingly, using the prosthesis did not improve navigational precision in the path reproduction task, where patients could rely on their enhanced non-visual sensory information instead. Three of four patients actually showed increased imprecision when navigating with vision on this task. However, in the triangle completion task for which vision is more relevant and patients showed higher non-visual errors (compared to in the path reproduction task), patients showed differing results, with two of four showing similar improvements in precision as controls when using vision.

In summary, the visual information afforded by the retinal prosthesis did not consistently improve performance on both tasks. This is partly because the visual signal is different to native vision and involves using effortful strategies to estimate angles and distances, but also partly because patients were expert non-visual navigators in these tasks, and so the non-visual information was much more reliable than the visual information provided by the prosthesis. Enhanced non-visual performance in these patients may be the result of increased practice and/or cortical reorganisation (e.g. [30, 31, 32]). It is possible that given more complex paths, with more turns and greater distances, non-visual information would become so much less reliable that patients could demonstrate larger benefits from the new visual signal. Additionally, although patients completed training covering how to use the device on receiving the implant, all four patients in this study reported that they tended not to use the prosthesis for navigation in everyday life, having learnt to rely on non-visual navigational strategies, (all four patients had been blind for at least 15 years). Whilst in this study, we found that neither controls nor patients learnt over the course of the experiment, it would be interesting to assess whether with more trials and feedback, patients could improve their navigational precision, as they learnt to compute distance and angle information with the new visual signal. Similarly, patients who have been visually deprived for a shorter amount of time, may show less-developed non-visual sensory skills, increased reliance on the sensory signal afforded by the prosthesis in daily activities, and consequently possibly improved performance on multisensory tasks when the prosthetic visual signal is available.

## Conclusion

Patients implanted with the Argus II retinal prosthesis did not use the vision afforded by the prosthesis to reduce their navigational uncertainty for path reproduction, but two patients did show a similar multisensory benefit to normally sighted adults in the triangle completion task. All patients showed greater precision than controls in both tasks when navigating without vision. The differential results between patients and control participants may be partly accounted for by differences in (i) the reliability of the visual signal afforded by the prosthesis and the control-worn goggles, and (ii) the reliability of non-visual processing between groups, due to the duration of visual deprivation, practice and/or sensory reorganisation.

## Supporting Information

S1 Fig Graph showing variable errors (A) or constant errors (B) with vision against errors without vision. (EPS)

S2 Fig Graph showing mean trajectories walked on both tasks, by normally sighted adults (A) and patients (B). (EPS)

S1 Table Results of a repeated measures ANOVA comparing variable and constant errors between conditions (prosthetic vision system-on, residual vision system-off, no vision system-off and blindfolded). There were no significant differences in variable errors or constant errors between conditions.
(EPS)

S2 Table Variable errors (m) without vision and with vision, for path reproduction and triangle completion tasks. (EPS)

S3 Table Constant errors (m) without vision and with vision, in path reproduction and triangle completion tasks. (EPS)

S4 Table Paired t-test results of effect of block number on variable error, and linear regression results of effect of trial number on constant error, for each condition within each task. (EPS)

## References

[1] Gallistel CR. Representations in animal cognition: an introduction. Cognition. 1990;37(1–2):1–22. Epub 1990/11/01. . 2269003 10.1016/0010-0277(90)90016-d

[2] Morris RGM. Spatial localization does not require the presence of local cues. Learning and Motivation. 1981;12(2):239–60. 10.1016/0023-9690(81)90020-5

[3] Mittelstaedt ML, Mittelstaedt H. Homing by path integration in a mammal. Naturwissenschaften. 1980;67(11):566–7. 10.1007/BF00450672

[4] Burgess N. Spatial Cognition and the Brain. Annals of the New York Academy of Sciences. 2008;1124(1):77–97. 10.1196/annals.1440.002 18400925

[5] Bates SL, Wolbers T. How cognitive aging affects multisensory integration of navigational cues. Neurobiology of Aging. 2014;(0). 10.1016/j.neurobiolaging.2014.04.003 24952995

[6] Kalia AA, Schrater PR, Legge GE. Combining Path Integration and Remembered Landmarks When Navigating without Vision. PloS one. 2013;8(9):e72170 10.1371/journal.pone.0072170 24039742 PMC3764103

[7] Nardini M, Jones P, Bedford R, Braddick O. Development of cue integration in human navigation. Current biology: CB. 2008;18(9):689–93. Epub 2008/05/03. 10.1016/j.cub.2008.04.021 . 18450447

[8] Tcheang L, Bulthoff HH, Burgess N. Visual influence on path integration in darkness indicates a multimodal representation of large-scale space. Proceedings of the National Academy of Sciences of the United States of America. 2011;108(3):1152–7. Epub 2011/01/05. 10.1073/pnas.1011843108 ; PubMed Central PMCID: PMCPmc3024704. 21199934 PMC3024704

[9] Petrini K, Remark A, Smith L, Nardini M. When vision is not an option: children's integration of auditory and haptic information is suboptimal. Developmental science. 2014;17(3):376–87. 10.1111/desc.12127 24612244 PMC4240463

[10] Proulx MJ, Brown DJ, Pasqualotto A, Meijer P. Multisensory perceptual learning and sensory substitution. Neuroscience and biobehavioral reviews. 2014;41:16–25. Epub 2012/12/12. 10.1016/j.neubiorev.2012.11.017 . 23220697

[11] Chebat DR, Maidenbaum S, Amedi A. Navigation using sensory substitution in real and virtual mazes. PloS one. 2015;10(6):e0126307 Epub 2015/06/04. 10.1371/journal.pone.0126307 ; PubMed Central PMCID: PMCPmc4454637. 26039580 PMC4454637

[12] Alary F, Duquette M, Goldstein R, Elaine Chapman C, Voss P, La Buissonnière-Ariza V, et al Tactile acuity in the blind: A closer look reveals superiority over the sighted in some but not all cutaneous tasks. Neuropsychologia. 2009;47(10):2037–43. 10.1016/j.neuropsychologia.2009.03.014 19467354

[13] Goldreich D, Kanics IM. Tactile acuity is enhanced in blindness. The Journal of neuroscience: the official journal of the Society for Neuroscience. 2003;23(8):3439–45. Epub 2003/04/30. . 12716952 10.1523/JNEUROSCI.23-08-03439.2003 PMC6742312

[14] Lessard N, Pare M, Lepore F, Lassonde M. Early-blind human subjects localize sound sources better than sighted subjects. Nature. 1998;395(6699):278–80. Epub 1998/09/29. 10.1038/26228 . 9751055

[15] Voss P, Lassonde M, Gougoux F, Fortin M, Guillemot JP, Lepore F. Early- and late-onset blind individuals show supra-normal auditory abilities in far-space. Current biology: CB. 2004;14(19):1734–8. Epub 2004/10/02. 10.1016/j.cub.2004.09.051 . 15458644

[16] Fortin M, Voss P, Lord C, Lassonde M, Pruessner J, Saint-Amour D, et al Wayfinding in the blind: larger hippocampal volume and supranormal spatial navigation. Brain: a journal of neurology. 2008;131(Pt 11):2995–3005. Epub 2008/10/16. 10.1093/brain/awn250 . 18854327

[17] Merabet LB, Pascual-Leone A. Neural reorganization following sensory loss: the opportunity of change. Nat Rev Neurosci. 2010;11(1):44–52. 10.1038/nrn2758 19935836 PMC3898172

[18] Ptito M, Fumal A, de Noordhout AM, Schoenen J, Gjedde A, Kupers R. TMS of the occipital cortex induces tactile sensations in the fingers of blind Braille readers. Experimental brain research Experimentelle Hirnforschung Experimentation cerebrale. 2008;184(2):193–200. Epub 2007/08/25. 10.1007/s00221-007-1091-0 . 17717652

[19] Humayun MS, Dorn JD, da Cruz L, Dagnelie G, Sahel J-A, Stanga PE, et al Interim Results from the International Trial of Second Sight's Visual Prosthesis. Ophthalmology. 2012;119(4):779–88. 10.1016/j.ophtha.2011.09.028 22244176 PMC3319859

[20] Brainard DH. The Psychophysics Toolbox. Spatial vision. 1997;10(4):433–6. Epub 1997/01/01. . 9176952

[21] Pelli DG. The VideoToolbox software for visual psychophysics: transforming numbers into movies. Spatial vision. 1997;10(4):437–42. Epub 1997/01/01. . 9176953

[22] Rousseeuw PJ, Driessen Kv. A Fast Algorithm for the Minimum Covariance Determinant Estimator. Technometrics. 1999;41(3):212–23. 10.2307/1270566

[23] Verboven S, Hubert M. LIBRA: a MATLAB library for robust analysis. Chemometrics and Intelligent Laboratory Systems. 2005;75(2):127–36. 10.1016/j.chemolab.2004.06.003

[24] Cheng K, Shettleworth SJ, Huttenlocher J, Rieser JJ. Bayesian integration of spatial information. Psychological bulletin. 2007;133(4):625–37. 10.1037/0033-2909.133.4.625 17592958

[25] Ernst MO. A Bayesian view on multimodal cue integration In: Knoblich G, editor. Human Body Perception From The Inside Out. Oxford, UK: Oxford University Press; 2006 p. 105–31.

[26] Perez Fornos A, Sommerhalder J, da Cruz L, Sahel JA, Mohand-Said S, Hafezi F, et al Temporal properties of visual perception on electrical stimulation of the retina. Investigative ophthalmology & visual science. 2012;53(6):2720–31. Epub 2012/03/27. 10.1167/iovs.11-9344 . 22447863

[27] Petrini K, Caradonna A, Foster C, Burgess N, Nardini M. The visual influence on path reproduction in darkness is stronger during childhood. Journal of vision. 2014;14(10):1345 10.1167/14.10.1345

[28] Loomis JM, Klatzky RL, Golledge RG, Cicinelli JG, Pellegrino JW, Fry PA. Nonvisual navigation by blind and sighted: assessment of path integration ability. Journal of experimental psychology General. 1993;122(1):73–91. . 8440978 10.1037//0096-3445.122.1.73

[29] Foo P, Warren WH, Duchon A, Tarr MJ. Do humans integrate routes into a cognitive map? Map- versus landmark-based navigation of novel shortcuts. Journal of experimental psychology Learning, memory, and cognition. 2005;31(2):195–215. Epub 2005/03/10. 10.1037/0278-7393.31.2.195 . 15755239

[30] Weaver KE, Stevens AA. Attention and sensory interactions within the occipital cortex in the early blind: an fMRI study. Journal of cognitive neuroscience. 2007;19(2):315–30. Epub 2007/02/07. 10.1162/jocn.2007.19.2.315 . 17280519

[31] Lewald J. Exceptional ability of blind humans to hear sound motion: implications for the emergence of auditory space. Neuropsychologia. 2013;51(1):181–6. Epub 2012/11/28. 10.1016/j.neuropsychologia.2012.11.017 . 23178211

[32] Renier LA, Anurova I, De Volder AG, Carlson S, VanMeter J, Rauschecker JP. Preserved functional specialization for spatial processing in the middle occipital gyrus of the early blind. Neuron. 2010;68(1):138–48. Epub 2010/10/06. 10.1016/j.neuron.2010.09.021 20920797 PMC2951740

